# Widening disparities in health between educational levels and their determinants in later life: evidence from a nine-year cohort study

**DOI:** 10.1186/s12889-018-5181-7

**Published:** 2018-02-23

**Authors:** Takashi Oshio

**Affiliations:** Institute of Economic Research, Hitotsubashi University, 2-1 Naka, Kunitachi, Tokyo, 186-8603 Japan

**Keywords:** Disparities in health, Educational levels, Functional limitations, Self-rated health, Psychological distress, Mediation analysis

## Abstract

**Background:**

Education has attracted more attention as a key determinant of health in later life. In this study, the hypothesis that widened educational disparities in health can be observed in later life was investigated, and the factors that mediated the association between education and changes in health were also assessed.

**Methods:**

Using the 9-year (10-wave) longitudinal data of 20,024 individuals (9320 men and 10,704 women) aged 50–59 years at baseline, collected from a nationwide population survey in Japan (2005–2014), the changes in self-rated health, functional limitations, and psychological distress between educational levels were compared. Mediation analysis was further conducted to assess the factors that mediated the association between education and changes in health, with reference to six types of potential mediators (household spending, social participation, leisure-time physical activity, smoking, problem drinking, and regular health check-ups). The analyses were conducted separately for men and women.

**Results:**

All three health variables rapidly deteriorated among lower-educated men and women. For men, the six potential mediators mediated 55.2%, 64.3%, and 47.3% of the associations between educational levels and changes in self-rated health, functional limitations, and psychological distress, respectively. The proportions for women were 42.0%, 49.5%, and 58.8%, respectively. Social participation was the primary mediator, followed by physical activity, regular health check-ups, and smoking. In general, no substantial or consistent differences were observed between men and women.

**Conclusions:**

The results suggested that policy measures that encourage social participation and promote healthy behaviors can improve educational disparities in health in later life.

## Background

Education as a key determinant of health in later life has attracted more attention because it is one of the most stable indicators of one’s socioeconomic status after young adulthood [1, 2]. Education is also likely to affect other aspects of socioeconomic status that are associated with health [3, 4]. A well-established view is that health differences between educational levels increase with age. Health is predicted to deteriorate more rapidly with age for lower-educated individuals than for higher-educated individuals, which is known as the cumulative disadvantage hypothesis [5]. In line with this hypothesis, several studies have demonstrated that educational level is a key determinant of health disparities in later life among other several aspects of health, including mortality, disability, frailty, chronic diseases, mental health, self-rated health, or other health variables [5–13].

However, two key challenges must be addressed for the further understanding of the association between educational levels and health. First, more information is needed about the long-term changes in health at an individual level, particularly if the focus is on how health disparities will accumulate with age over time. Previous studies have often been based on cross-sectional or repeated cross-sectional data [8, 12, 13], and even if longitudinal data were used, analyses have often been limited to comparisons between a couple of survey waves with relatively short intervals [5–7, 10, 11, 14], with a recent exception that used longer longitudinal data [9]. Further evidence based on large-scale and extended longitudinal data must be obtained to examine the validity of the cumulative disadvantage hypothesis at an individual level [7].

Second and more importantly, the mechanism that explains the relationship between educational levels and widening health disparities with age has not been fully elucidated. Numerous studies have examined the possible explanations for the general relationship between education and health [3, 15]. In addition, results showed several potential mediators of this relationship. For example, a lower educational level is likely to cause material disadvantages, particularly in terms of income, which can reduce the access to healthy food and the chances of living in healthy conditions [16, 17]. Lower-educated individuals may also undertake an unhealthy lifestyle or behaviour, resulting in higher risks of worsening health [4, 18]. In this respect, how health behaviours and lifestyle habits, such as leisure-time physical activity, smoking, and problem drinking can link education to health should be assessed. In addition, social participation may be a potential mediator if it is positively associated with educational level [14], given that studies have demonstrated a positive association between social participation and health [19, 20]. However, existing observations about these mediating effects are conflicting, and as suggested by Chandola et al. [21], multiple pathways that link education and health must be considered, rather than focusing on a single potential mediator.

To address these challenges and further understand the association between educational levels and health, the validity of the cumulative disadvantage hypothesis was assessed at an individual level, and the factors that mediated the association between education and changes in health were investigated. For these purposes, the 10-wave (9-year) longitudinal data, obtained from a nationwide social survey, of 20,024 individuals (9320 men and 10,704 women) aged 50–59 years at baseline were used. The changes in health and its evolution over the 9-year period were compared between the lower- and middle−/higher-educated individuals, with a focus on three health variables (self-rated health [SRH], functional limitations, and psychological distress).

Furthermore, the factors that mediated the association between educational levels and changes in health over the 9-year period were assessed. Six potential mediators were considered: household spending (as an alternate for income), social participation, leisure-time physical activity, smoking, problem drinking, and regular health check-ups. A mediation analysis was conducted to evaluate the mediating effect of each of these six variables on the association between education and health.

All of these analyses were conducted separately for men and women. It might be possible that educational differences in health may increase differently among men and women, and that the mediating mechanisms might operate in different ways by gender.

## Methods

### Study sample

Data from the Longitudinal Survey of Middle-Aged and Older Adults, a nationwide 9-year (10-wave) panel survey, that was conducted by the Japanese Ministry of Health, Labour and Welfare (MHLW) each year between 2005 and 2014, were obtained. Samples in the first wave were collected nationwide in November 2005 through a two-stage random sampling procedure. First, 2515 districts were randomly selected from 5280 districts used in the MHLW’s nationwide, population-based “Comprehensive Survey of the Living Conditions of People on Health and Welfare,” which was conducted in 2004. The 5280 districts were, in turn, randomly selected from about 940,000 national census districts. Second, 40,877 residents aged 50–59 years as of October 30, 2005 were randomly selected from each selected district, according to its population size. A total of 34,240 individuals responded (response rate: 83.8%). The second to tenth waves of the survey were conducted in early November of each year from 2006 to 2014, and 22,748 individuals remained until the tenth wave (with an average attrition rate of 4.0% in each wave). No new respondents were added after the first wave.

Data of the 20,024 individuals (9320 men and 10,704 women), who participated for 9 years were used, and all information required in the present study were provided. The respondents were divided into lower-educated individuals (whose educational attainment was below high school, that is, less than 12 years of schooling in total) and middle−/higher-educated ones (who had graduated from high school or above). Lower-educated individuals, including those who had not completed high school, comprised 15.4% of the entire sample. The study sample consisted of 58.4% of the individuals who participated in the first wave. The key attributes between this study sample and dropouts were compared to assess the potential bias in the estimation results.

### Measurements

#### Health variables

Three health variables were considered: SRH, functional limitations, and psychological distress. SRH has often been used as a comprehensive alternative for general health conditions in social epidemiology because it has been repeatedly found to be a valid predictor of health outcomes, including mortality, physical and cognitive functioning, and morbidity [22–24]. In terms of SRH, the respondents were asked to choose 1 (*very good*), 2 (*good*), 3 (s*omewhat good*), 4 (*somewhat poor*), 5 (*poor*), or 6 (*very poor*) regarding their current health condition.

These categorical answers were used as a continuous variable with higher values that indicate poorer SRH. In terms of functional limitations, the respondents were asked whether they had any difficulty in each of the 10 activities of daily living (walking, getting out of bed, getting in/out of a chair, dressing, washing their face and hands, eating, toileting, bathing, ascending and descending stairs, and carrying purchased items). The degrees of functional limitations were also evaluated using the sum of items in which the respondents had difficulty performing.

Kessler 6 (K6) scores were established to measure psychological distress [25, 26]. From the survey, the respondents’ assessments of psychological distress were first obtained using a 6-item psychological distress questionnaire—“During the past 30 days, about how often did you feel a) nervous, b) hopeless, c) restless or fidgety, d) so depressed that nothing could cheer you up, e) that everything was an effort, and f) worthless?” The questionnaire was rated on a 5-point scale (0 = *none of the time* to 4 = *all of the time*). The sum of the reported scores were then calculated (range: 0–24) and defined as the K6 score. Higher K6 scores reflected higher levels of psychological distress.

#### Potential mediators

Six types of potential mediators were considered for the association between education and health (household spending, social participation, leisure-time physical activity, smoking, alcohol drinking, and regular health check-ups). Each variable was completely evaluated throughout the 10 waves. Household spending was considered as a key factor that represents the material conditions rather than income because the number of respondents who did not report household spending was significantly lower compared to those who did not own a household or have their own income and because dependent wives who did not work outside their house did not have income. Reported household spending throughout the 10 waves were summarized, and a binary variable of low household spending was established by allocating one to the lowest tertile of the sum and zero if otherwise.

In terms of social participation, respondents were asked whether they participated in 6 types of social participation (hobbies or cultural activities, exercise or sports, community events, support for children, support for the elderly, and other activities) within the past year from the date of the survey. The answers regarding social participation were summarized, showing that the respondents were engaged in each wave throughout the 10 waves (range: 0–60), and a binary variable of low social participation was established by allocating one to the lowest tertile of the sum and zero if otherwise.

Physical activity, smoking, alcohol drinking, and regular health check-ups were considered as key behaviours that are associated with health. Respondents were asked how they were engaged in leisure-time physical activity. A binary variable of low physical activity was then established by allocating one to those who did not engage in moderate (without breathlessness or heart palpitations) or more intense exercise at least few days per week throughout the 10 waves. The respondent was considered as a smoker if he/she answered that he/she was currently smoking all throughout the waves. Problem drinking was defined as an intake of more than two *go* (360 ml) per day of Japanese *sake* or an equivalent amount of alcohol, which corresponds to about 40 g of pure alcohol. This threshold was based on a study showing that maintaining alcohol consumption below 46 g/day minimized the risks of mortality in a Japanese population [27]. Those who drank above this threshold in at least one wave were considered as problem drinkers. Lastly, a binary variable for those with no regular health check-ups was established by allocating one to those who reported that they did not have a health check-up in at least one wave. In addition to these variables, binary variables of sex and each age (50–59 years old) at baseline and the baseline values of each health variable as covariates were used.

#### Statistical analyses

For the descriptive analysis, the baseline values and changes in the three health variables over the 9-year period between the lower- and middle−/higher-educated individuals were compared for both men and women. Then, two types of linear regression models (Models A and B) were estimated separately for men and women to explain the change in each health variable between baseline and each wave, allowing random effects to consider error terms at an individual level. In Model A, the wave was used as a continuous variable and the binary variable of low educational level as key explanatory variables, along with the covariates. The coefficients of the wave and low educational level were both expected to be positive. In Model B, the interaction term of the wave and low educational level were added to Model A. The coefficient of this interaction term was expected to be positive if low educational level adds to the pace of deterioration in health with increasing age.

In these regression models, each health variable was normalised by its mean and standard deviation to help assess and compare the substantive degrees of association between health and other variables. In addition, inverse probability weighting was used to mitigate potential sources of attrition bias [28, 29]. Specifically, the probit model was first estimated to predict observation presence through wave 10, using the baseline values of each health variable and the binary variables of lower education and each age at baseline. Then, the inverse of the predicted probability of presence was used as the weight when estimating the regression models.

Subsequently, a mediation analysis was performed separately for men and women, with the conventional three-step estimation procedure along with bootstrapping to assess the significance of the mediating effects [30, 31]. Changes in the three health variables over the 9-year period were the focus. In the first step, Model 1 was used to explain the change in each health variable between baseline and the tenth wave by the binary variable of low educational level. In the second step, Model 2 was used to explain each potential mediator by the binary variable of low educational level. In the third step, Model 3 was utilized to explain the change in each health variable by low educational level. In each of Models 1, 2, and 3, health variables at baseline as well as other covariates were controlled for.

For each potential mediator an actual mediator was suspected if the estimated coefficients of low educational level in Models 1 and 2 and the estimated coefficients for the potential mediators were all statistically significant. To examine the statistical significance of the mediating effect, the 95% confidence interval (CI) of the proportion of the association between education and the change in each health variable were subsequently estimated via bootstrap estimation with 2000 replications.

## Results

### Widening disparities in health

The first half of Table 1 shows the comparison (1) of the values at baseline and (2) the changes over the 9-year period for the lower- and middle−/higher-educated individuals in terms of SRH, functional limitations, and psychological distress, between lower- and middle−/higher-educated individuals. For both men and women, SRH and functional limitations at baseline were worse among lower-educated individuals than middle−/higher-educated ones, whereas no difference was observed in terms of psychological distress. Over the 9-year period, self-rated health and psychological distress deteriorated among lower-educated men, while functional limitations and psychological distress deteriorated among lower-educated women. Deterioration in functional limitations or self-rated health showed no difference among men and women with varying educational backgrounds. However, it should be noted that baseline values of health variables or other covariates were not controlled for in Table 1.

**Table 1:** Comparing health and other variables by educational level^a^ (*N* = 20,024)

Educational level	Men (*n* = 9320)				Women (*n* = 10,704)			
	Lower	Middle/higher	Difference		Lower	Middle/higher	Difference	
	(A)	(B)	A-B	*p*-value	(A)	(B)	A-B	*p*-value
Health variables								
(1) Values at baseline								
Self-rated health	2.84	2.68	0.16	< 0.001	2.91	2.68	0.23	< 0.001
(range: 1-6)	(0.97)	(0.94)			(0.96)	(0.90)		
Functional limitations	0.22	0.14	0.08	0.007	0.32	0.21	0.11	< 0.001
(range: 0-10)	(1.27)	(0.99)			(1.44)	(1.07)		
Psychological distress	2.66	2.72	−0.06	0.584	3.16	3.05	0.11	0.301
(range: 0-24)	(3.71)	(3.77)			(4.04)	(3.82)		
(2) Changes over ten waves								
Self-rated health	0.21	0.15	0.06	0.042	0.16	0.14	0.02	0.404
	(1.05)	(1.00)			(1.00)	(0.94)		
Functional limitations	0.27	0.24	0.03	0.505	0.47	0.26	0.21	< 0.001
	(1.94)	(1.66)			(2.19)	(1.72)		
Psychological distress	0.58	0.05	0.53	< 0.001	0.60	0.37	0.23	0.029
	(4.22)	(3.85)			(4.21)	(3.95)		
Potential mediators over ten waves								
Low household spending^b^	0.45	0.34	0.11	< 0.001	0.49	0.37	0.12	< 0.001
	(0.50)	(0.47)			(0.50)	(0.48)		
Low social activity^b^	0.53	0.30	0.24	< 0.001	0.53	0.28	0.24	< 0.001
	(0.50)	(0.46)			(0.50)	(0.45)		
Low physical activity	0.98	0.95	0.04	< 0.001	0.97	0.94	0.03	< 0.001
	(0.14)	(0.23)			(0.18)	(0.24)		
Smoking	0.61	0.51	0.10	< 0.001	0.29	0.19	0.10	< 0.001
	(0.49)	(0.50)			(0.46)	(0.40)		
Problem drinking	0.19	0.17	0.02	0.022	0.12	0.10	0.02	0.063
	(0.39)	(0.37)			(0.32)	(0.30)		
No regular health check-up	0.80	0.66	0.14	< 0.001	0.82	0.75	0.06	< 0.001
	(0.40)	(0.48)			(0.39)	(0.43)		
Number of individuals	1452	7868			1631	9073		

^a^ Figures in the parentheses are standard deviations. ^b^ Lowest tertile

The second half of Table 1 shows the comparison of the six potential mediators in terms of educational level over the 9-year period. Lower-educated individuals were at significantly higher risks of low household spending, low social participation, low physical activity, smoking, problem drinking, and no regular check-ups compared to middle−/higher-educated individuals, while the difference in the proportion of problem drinking was small for both men and women and significant only at the 10% level for women.

To confirm the widening educational disparities in health with age, Table 2 presents the estimation results of the regression models to explain the change in each health variable between baseline and each wave after controlling for sex and age at baseline. In Model A, results showed that, for both men and women, low educational level accelerated deterioration in health. This result was obtained even after controlling for (i) the adverse effect of aging on health (which is captured by a positive coefficient of a continuous variable of the wave), and (ii) the initial level effect (which means that higher initial levels reduced additional increases in subsequent waves and is indicated by a negative coefficient of the health variable at baseline).

**Table 2:** Estimated associations with changes in normalised health variables between baseline and each wave^a^

	Men (*n* = 9320 × 10 waves)				Women (*n* = 10,704 × 10 waves)			
	Model A		Model B		Model A		Model B	
	Coef. ^b^	SE^c^	Coef.	SE	Coef.	SE	Coef.	SE
(1) Self-rated health^d^								
Low-educated	0.116^***^	(0.012)	0.081^***^	(0.015)	0.105^***^	(0.011)	0.092^***^	(0.014)
Low-educated × Wave			0.006^***^	(0.002)			0.002	(0.002)
Wave	0.017^***^	(0.001)	0.016^***^	(0.001)	0.015^***^	(0.001)	0.014^***^	(0.001)
Self-rated health at baseline	−0.467^***^	(0.004)	−0.467^***^	(0.004)	−0.474^***^	(0.004)	−0.474^***^	(0.004)
(2) Functional limitations^d^								
Low-educated	0.039^***^	(0.011)	0.020	(0.015)	0.089^***^	(0.012)	0.029	(0.016)
Low-educated × Wave			0.004^*^	(0.002)			0.011^***^	(0.002)
Wave	0.015^***^	(0.001)	0.015^***^	(0.001)	0.019^***^	(0.001)	0.017^***^	(0.001)
Functional limitations at baseline	−0.538^***^	(0.005)	−0.538^***^	(0.005)	−0.440^***^	(0.005)	−0.440^***^	(0.005)
(3) Psychological distress^d^								
Low-educated	0.092^***^	(0.012)	0.018	(0.014)	0.052^***^	(0.012)	0.021	(0.015)
Low-educated × Wave			0.014^***^	(0.002)			0.006^***^	(0.002)
Wave	−0.001	(0.001)	−0.003^***^	(0.001)	0.006^***^	(0.001)	0.005^***^	(0.001)
Psychological distress at baseline	−0.431^***^	(0.005)	−0.431^***^	(0.005)	−0.380^***^	(0.005)	−0.380^***^	(0.005)

^a^Controlled for ages at baseline. ^b^ Coefficient. ^c^ Standard error. ^d^ Normalised by mean and standard deviation

^***^p < 0.001, ^*^p < 0.05

By adding the interaction term between low educational level and the wave in Model B, the coefficients of the interaction terms were positive and significant in all models, except for self-rated health for women. This observation indicates that low educational level generally accelerated the deterioration in health with age for both men and men.

### Mediation analysis

The estimation results of Models 1 and 3 based on the mediation analysis are presented in Table 3, which focuses on the change in health variables between baseline and the tenth wave. The results of Model 1 confirmed the adverse effect of low educational level on the changes in all the three types of health variables. The results of Model 2 are not presented to conserve space (available upon request), but it was confirmed that all potential mediators were significantly associated with low educational level (*p* <  0.001).

**Table 3:** Estimated associations with changes in normalised health variables over the 9-year period^a^

	Men (*n* = 9320)				Women (*n* = 10,704)			
	Model 1		Model 3		Model 1		Model 3	
	Coef.^b^	SE^c^	Coef.	SE	Coef.	SE	Coef.	SE
(1) Self-rated health^d^								
Low-educated	0.16^***^	(0.02)	0.08^***^	(0.02)	0.17^***^	(0.02)	0.11^***^	(0.02)
Low household spending			0.02	(0.02)			−0.05^***^	(0.01)
Low social participation			0.23^***^	(0.02)			0.21^***^	(0.02)
Low physical activity			0.23^***^	(0.04)			0.21^***^	(0.03)
Smoking			0.05^**^	(0.02)			0.03	(0.02)
Problem drinking			0.05^**^	(0.02)			−0.04	(0.02)
No regular health check-up			0.07^***^	(0.02)			0.04^**^	(0.02)
Self-rated health at baseline	−0.55^***^	(0.01)	−0.57^***^	(0.01)	−0.58^***^	(0.01)	−0.59^***^	(0.01)
(2) Functional limitations^d^								
Low-educated	0.05^*^	(0.02)	−0.01	(0.03)	0.16^***^	(0.03)	0.10^***^	(0.03)
Low household spending			0.05^*^	(0.02)			−0.03	(0.02)
Low social participation			0.20^***^	(0.02)			0.19^***^	(0.02)
Low physical activity			0.13^**^	(0.05)			0.14^***^	(0.04)
Smoking			0.01	(0.02)			0.13^***^	(0.02)
Problem drinking			0.08^**^	(0.03)			−0.05	(0.03)
No regular health check-up			0.02	(0.02)			0.11^***^	(0.02)
Functional limitations at baseline	−0.58^***^	(0.01)	−0.58^***^	(0.01)	−0.50^***^	(0.01)	−0.51^***^	(0.01)
(3) Psychological distress^d^								
Low-educated	0.14^***^	(0.02)	0.07^***^	(0.02)	0.09^***^	(0.02)	0.04	(0.02)
Low household spending			0.03^*^	(0.02)			−0.01	(0.01)
Low social participation			0.19^***^	(0.02)			0.15^***^	(0.02)
Low physical activity			0.08^*^	(0.03)			0.06^*^	(0.03)
Smoking			0.00	(0.01)			0.04^*^	(0.02)
Problem drinking			0.05^*^	(0.02)			0.00	(0.02)
No regular health check-up			0.06^***^	(0.02)			0.07^***^	(0.02)
Psychological distress at baseline	−0.52^***^	(0.01)	−0.54^***^	(0.01)	−0.47^***^	(0.01)	− 0.48^***^	(0.01)

^a^Controlled for ages at baseline. ^b^ Coefficient. ^c^ Standard error. ^d^ Normalised by mean and standard deviation

^***^ *p* < 0.001, ^**^ *p* < 0.01, ^*^p  < 0.05

The results of Models 3 help understand the mediating mechanism. For example, in the case of SRH for men, the estimated coefficient of low educational level was substantially attenuated to 0.08 from 0.16 in Model 1, after controlling for the six potential mediators, suggesting that a substantial portion of the association between education and the change in SRH was influenced by those mediators. Among the six variables, low social participation, smoking, problem drinking, and no regular health check-ups were positively associated with deteriorated SRH. Household spending was not related to SRH. A reduction in the estimated coefficient of low educational level from Model 1 to 3 was commonly observed in all models, while the levels of the coefficient were somewhat different between men and women. Another finding was that estimation results of the six mediators were not much different between men and women in terms of the magnitudes and statistical significance of their estimated coefficients; notably, low social participation and physical activity were most closely associated with the changes in health variables in all models.

Table 4 shows the comparison of the magnitude of each variable’s mediating effects as well as statistical significance. In the case of men’s self-rated health, social participation had the largest mediating effect, which accounted for 31.1% of the association between educational levels and SRH. The magnitude of the mediating effect of social participation was remarkably higher than that of physical activity (15.0%), regular health check-ups (6.0%), and smoking (3.1%). The mediating effects of these four variables were all significant, given that the bootstrap-estimated 95% CI did not include zero. By contrast, the mediating effect of household spending or problem drinking was not significant. The mediating effect of these six potential mediators accounted for 57.3% (95% CI: 28.9-58.6%) of the association between low educational level and SRH. If limited to four significant mediators, the mediating effect was 55.2% (95% CI: 44.2-66.1%) in total.

**Table 4:** Estimated proportions of the associations between education and health mediated by each mediator^a^ (*N* = 20,024)

	Men (*n* = 9320)		Women (*n* = 10,704)	
	Proportion (%)	95% CI^b^	Proportion (%)	95% CI
Self-rated health				
Low household spending	1.0	(−1.6, 3.5)	−3.6	(−6.1, − 1.2)
Low social participation	31.1^†c^	(24.4, 37.7)	30.7^†^	(24.3, 37.2)
Low physical activity	15.0^†^	(6.8, 23.2)	9.3^†^	(4.2, 14.4)
Smoking	3.1^†^	(0.5, 5.7)	2.0	(−1.2, 5.1)
Problem drinking	1.2	(−0.3, 2.6)	−0.8	(−2.2, 0.6)
No regular health check-up	6.0^†^	(2.2, 9.7)	2.0^†^	(0.1, 3.9)
Total	57.3^†^	(28.9, 85.6)	39.5^†^	(12.3, 66.8)
Total for significant mediators^d^	55.2^†^	(44.2, 66.1)	42.0^†^	(33.8, 50.1)
Functional limitations				
Low household spending	0.3	(−3.2, 3.8)	−2.3	(−5.7, 1.2)
Low social participation	41.8^†^	(32.3, 51.2)	27.4^†^	(18.7, 36.2)
Low physical activity	12.1^†^	(7.4, 16.8)	7.3^†^	(3.3, 11.3)
Smoking	3.9†	(0.3, 7.6)	9.8^†^	(−4.5, 15.1)
Problem drinking	0.8	(−1.0, 2.6)	−0.9	(−2.6, 0.9)
No regular health check-up	6.5†	(3.0, 10.0)	4.9^†^	(2.0, 7.9)
Total	65.4^†^	(21.3, 109.5)	46.4^†^	(4.3, 88.4)
Total for significant mediators^d^	64.3^†^	(53.6, 74.9)	49.5^†^	(38.8, 60.3)
Psychological distress				
Low household spending	2.4	(−0.8, 5.5)	−1.6	(−6.3, 3.1)
Low social participation	32.2^†^	(24.1, 40.3)	46.2^†^	(33.2, 59.1)
Low physical activity	7.7^†^	(1.9, 13.5)	6.5^†^	(0.2, 12.8)
Smoking	0.6	(−2.4, 3.6)	4.7	(−1.9, 11.4)
Problem drinking	1.3	(−0.5, 3.1)	0.2	(−2.1, 2.5)
No regular health check-up	7.4^†^	(2.8, 11.9)	6.1^†^	(2.0, 10.3)
Total	51.6^†^	(16.0, 87.3)	62.1^†^	(7.9, 116.4)
Total for significant mediators^d^	47.3^†^	(36.9, 57.6)	58.8^†^	(44.9, 72.7)

^a^Controlled for baseline value of each health variable as well as ages at baseline

^b^95% confidence intervals obtained via bootstrap estimation with 2000 replications

^c^Indicates that bootstrap-estimated 95% CI was above zero

^d^Mediators with ^†^

Largely similar results were obtained for other combinations of the health variable and gender. For men, the six potential mediators accounted for 64.3% and 47.3% of functional limitations and psychological distress, respectively. The proportions for women were 42.0%, 49.5%, and 58.8% for the three health variables, respectively, not much different from those for men. For both men and women, social participation was the primary mediator for all health variables. Albeit to a lesser extent, leisure-time physical activity and regular health check-up, and smoking in some cases, were found to be important mediators for all health variables for both genders.

## Discussion

In the present study, the association between the changes in health and educational levels were investigated using the 10-wave longitudinal data of the individuals aged 50–59 years old at baseline. The estimation results clearly support the hypothesis that educational disparities in health would accumulate with age in terms of SRH, functional limitations, and psychological distress. These results were generally in accordance with those in previous studies that demonstrated educational disparities in health [5–13], although the present study additionally revealed the changes in disparities over the 9-year period.

The results of the mediating analysis highlighted the importance of the pathways that link education to health in later life. The proportions of the association between educational levels and the change in heath mediated by a set of six factors (household spending, social participation, leisure-time physical activity, smoking, problem drinking, and regular check-ups) were in the range from 47.3% to 64.3% and from 42.0% to 58.8% for men and women, respectively, depending on health variables (self-rated health, functional limitations, and psychological distress). These results suggested that we can construct policy measures to alleviate the accumulation of educational disparities in health by blocking the pathways that link low educational level to health.

In this respect, the key mediators for the association between education and health must be identified. Moreover, the prediction of health behaviors as key mediators is also important, as already suggested by previous studies [4, 18]. Indeed, estimation results confirmed that leisure-time physical activity and, to a lesser extent, smoking mediated the effect of education on health, whereas problem drinking did not. In addition, regular health check-up, which is not a narrowly defined heath behaviour, was also an important mediator. This result is also consistent with the assumption that health literacy mediates the effect of education on health [32] because it is reasonable to argue that individuals with a higher level of health literacy are more inclined to have regular heath check-ups.

Another significant finding is that social participation was the primary mediator of the association between education and health because of the magnitude of its mediating effect was well above those of other factors for both men and women. Numerous studies have demonstrated that social participation has a favourable effect on health [19, 20]. The results of the present study suggested that lower-educated individuals are at high risk in failing (or be reluctant) to engage in social participation, which in turn affects the health of lower-educated individuals.

However, a one-way causation from social participation (as well as other mediators) may not affect health. Rather, a two-way causation between the two variables may be assumed, considering that healthier individuals are more likely to engage intensively in social participation, which in turn further enhances their health. This two-way causation between social participation and health may result in the accumulation of the mediating effect of social participation over time. Compared to the present study, Ettman et al. [14] indicated a more limited mediating effect of social participation between educational levels on frailty. The difference was probably attributed to the difference in the time intervals in observing the change in health: 2 years in the study by Ettman et al. versus 9 years in the present study.

In contrast to social participation and health behaviours, a somewhat surprising result was that household spending, which was used as an alternative for income, did not have any mediating effect on the association between educational levels and health. Two remarks should be made on this result. First, it may probably be wrong to argue for a limited mediating effect of income, because income may likely provide material sources to health-promoting behaviours, access to health service, and healthy lifestyle. In this sense, income may possibly arbitrate the mediating effects of other factors. Second, the present study, which focused on how income (along with other factors) mediated the effects of education on health, did not address the differential effects of education versus income on health, which should be addressed in another analytic framework [1, 33].

Finally, the results did not show any substantial differences between men and women, and gender differences depended on the types of health variable. For both men and women, educational disparities in health widened at a largely similar pace, albeit somewhat differently across health variables. In addition, the proportion of the association between education and health mediated by six potential mediators was in the range from 47.3% to 64.3% and 42.0% to 58.8% for men and women, respectively, which were largely overlapped. Moreover, the key mediator was social participation for both men and women, and physical activity, regular health check-up, and smoking worked as important mediators commonly for both genders. However, we should be cautious in any generalization, because the results may depend on socio-institutional backgrounds.

The present study has several limitations. First, the educational difference in mortality was not analysed due to lack of data availability from the current dataset. Second, potential mediators for the association between education and health were not comprehensively explored, although their association was significantly mediated by the six factors that were considered in the present study. Hence, one should be cautious in interpreting the proportion of the mediated association in Table 4. The remaining proportion did not indicate the magnitude of the direct unmediated effect of education on health. Third and most importantly, the possibility that a third unobserved factor that affects both education and the mediators exist was not ruled out. For instance, some genetic characteristics or personality trait can make an individual more inclined to both continue his/her education and participate in social activities. If that is the case, caution should be undertaken in interpreting the observed association between education and the mediators as well as its effects on the association between education and health.

## Conclusions

Based on the statistical analyses using the 10-wave cohort data of the nationwide survey in Japan, educational disparities tended to widen with age in later life. In addition, a substantial portion of the associations between educational levels and changes in health was mediated by social participation and health-related activities, which contributed to a cumulative disadvantage of low educational level. These results suggested that policy measures that encourage social participation and promote healthy behaviours can improve educational disparities in health in later life.

## Abbreviations

K6: Kessler 6
SRH: Self-rated health
